# Depressive symptoms and occupational correlates among psychiatric nurses in northern Jiangsu: an explanatory sequential mixed-methods study

**DOI:** 10.3389/fpsyt.2026.1967611

**Published:** 2026-10-21

**Authors:** Yan Han, Jie He, Qian Wang, Yan Tang

**Affiliations:** 1 Department of Nursing, Huai’an No.3 People’s Hospital, Huaian Second Clinical College of Xuzhou Medical University, Huai’an, China; 2 Department of Bipolar Disorders, Huai’an No.3 People’s Hospital, Huaian Second Clinical College of Xuzhou Medical University, Huai’an, China; 3 Department of Intensive Psychiatry, Huai’an No.3 People’s Hospital, Huaian Second Clinical College of Xuzhou Medical University, Huai’an, China

**Keywords:** depressive symptoms, mixed methods, night shift, occupational strain, psychiatric nursing, psychological resilience, workplace violence

## Abstract

**Background:**

Psychiatric nurses manage aggression, interrupted recovery and emotionally demanding care. Surveys quantify distress but rarely show how these experiences accumulate during routine work.

**Aim:**

We aimed to estimate the prevalence of a positive depressive-symptom screen among psychiatric nurses and to quantify its associations with four prespecified occupational exposures: workplace violence, night-shift burden, occupational strain and psychological resilience. A longitudinal qualitative phase then examined how nurses experienced these same exposures during routine work.

**Methods:**

Four tertiary public psychiatric hospitals in northern Jiangsu participated from January 2025 to March 2026. A convenience sample of 357 nurses completed the survey. Workplace violence during the preceding 12 months was assessed with Wang’s Workplace Violence Scale; at least one physical, verbal or psychological, or sexual incident perpetrated by a psychiatric inpatient or visiting family member defined exposure. Modified Poisson regression with hospital fixed effects estimated prevalence ratios (PRs) for an SDS-positive screen. A purposive subsample of 20 nurses completed interviews, diaries and observation over three months. Colaizzi’s method and a joint display supported qualitative analysis and integration.

**Results:**

Of 357 nurses, 138 (38.7%) screened positive. Nurses reporting violence had a higher screening prevalence (PR 1.65, 95% CI 1.31–2.08). PRs per SD were 1.48 (95% CI 1.33–1.66) for night-shift burden and 1.45 (95% CI 1.28–1.65) for occupational strain. Resilience showed the opposite pattern (PR 0.79 per SD, 95% CI 0.70–0.88). Qualitative accounts centered on repeated strain, a value–recognition mismatch, unequal recovery resources and support considered generic or poorly timed.

**Conclusions:**

Depressive symptoms were common. Nurses identified practical targets: safer work organization, rapid post-incident contact and support tailored to individual circumstances. Cross-sectional estimates do not establish diagnosis or causation.

## 1 Introduction

Psychiatric nurses work at the center of therapeutic care. The job also brings unpredictable behavior, sustained emotional demands, secondary trauma and moral distress. Reviews report substantial burdens of burnout, compassion fatigue and secondary traumatic stress in this workforce (1, 2). Forensic mental health nurses may experience vicarious trauma, while acute mental health nurses report moral distress related to aggression, time pressure and patient safety (3, 4). Across nursing samples, burnout is also closely associated with depressive symptoms (5). These outcomes can affect staff well-being and workforce sustainability.

Some pressures are built into daily work. Workplace violence has physical and psychological consequences, including anxiety, post-traumatic stress and depressive symptoms (6). Mental health nurses in Italy describe lasting changes in vigilance, work habits and clinical practice after assault (7), and a multicenter longitudinal study of Chinese nurses linked exposure to subsequent depression and anxiety (8). De-escalation training, reflective practice and ward-level review may help manage conflict, although intervention evidence remains limited (9); international reviews of prevention programs converge on multicomponent approaches that combine training, post-incident debriefing, rapid response and environmental modification (10). Night and rotating shifts can disrupt sleep and recovery; a meta-analysis of nurses reported higher odds of depression among those working nights (11), and sleep problems are associated with burnout in nursing populations (12). Counts of major incidents cannot capture every threat, near miss or period of anticipatory vigilance. Staffing and ward responses may determine whether these experiences remain isolated or accumulate.

Recognition also matters. Mental health nurses often rely on informal social support (13). They describe a complex professional identity grounded in distinctive therapeutic skills (14), yet associative stigma can devalue the specialty (15). Resilience in mental health nursing is associated with better well-being and lower burnout or distress (16, 17). Resilience training and mindfulness interventions may improve resilience and some work-related outcomes, although effects vary across settings and measures (18, 19). Reviews of workplace interventions also indicate that job demands, organizational support and modifiable working conditions warrant system-level action (20–22). Treating resilience as the main remedy would leave preventable workplace hazards untouched.

The aim of this study was to estimate the prevalence of a positive depressive-symptom screen among psychiatric nurses in northern Jiangsu, to quantify its associations with four prespecified occupational exposures — workplace violence, night-shift burden, occupational strain and psychological resilience — and to use a three-month qualitative phase to explain how those exposures were experienced in routine practice. We used a multicenter survey to measure screening burden and these four occupational correlates. The study addressed three questions. How common and severe were depressive symptoms? How were violence, night shifts, strain and resilience related to screening status? What did nurses’ accounts add to those estimates?

## 2 Methods

### 2.1 Study design and setting

We used an explanatory sequential mixed-methods design (QUAN→qual) (**Figure 1**). The quantitative phase was a multicenter cross-sectional survey conducted from January 2025 to March 2026 in four tertiary public psychiatric hospitals located in Xuzhou, Lianyungang, Huai’an and Suqian, northern Jiangsu Province, China. A purposively selected subsample then entered a three-month qualitative follow-up. Integration occurred during sampling, interpretation and development of a joint explanatory framework.

**Figure 1:**
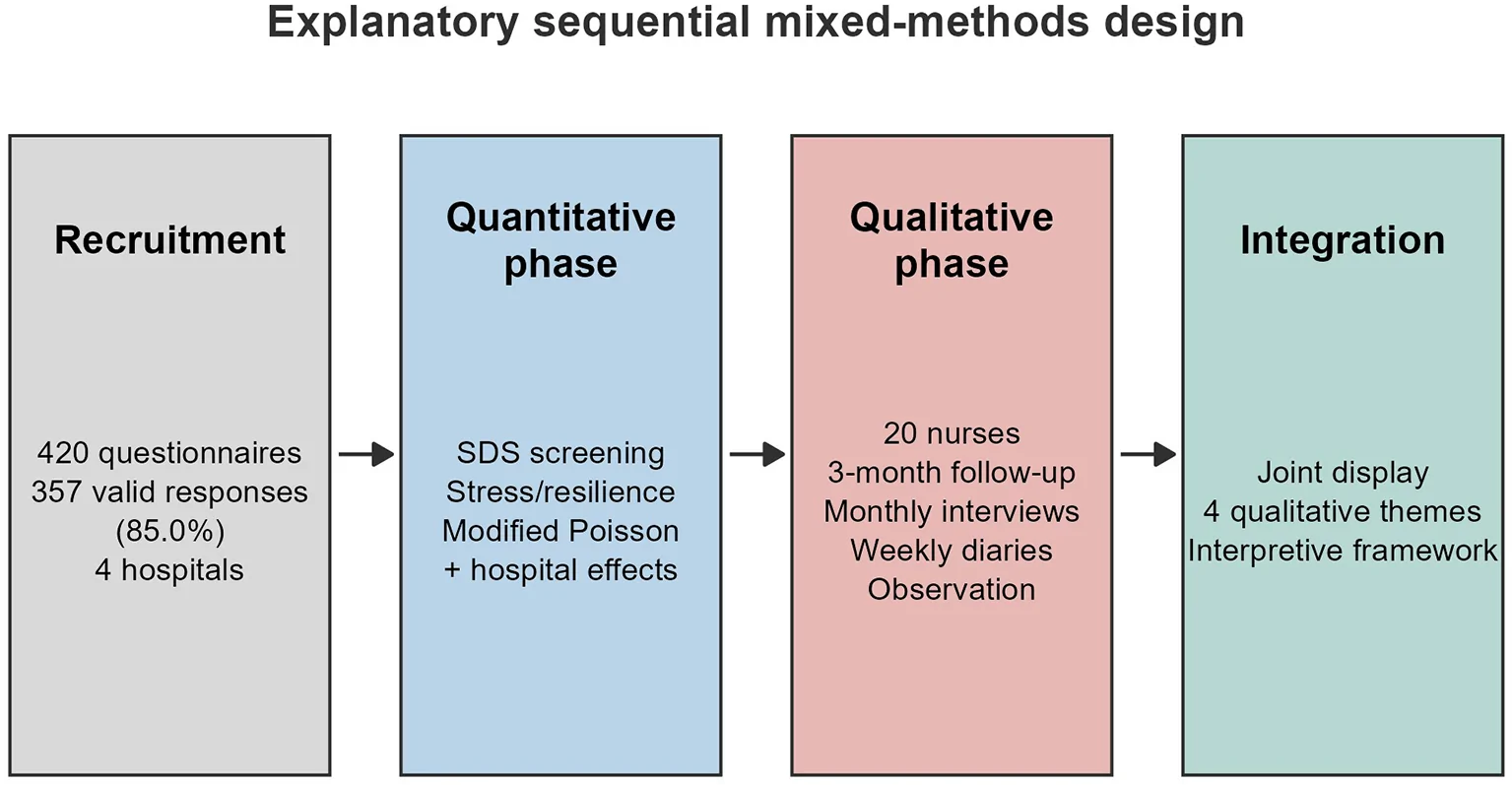
Explanatory sequential mixed-methods design. Quantitative survey data from 357 nurses informed purposive selection of 20 participants for a three-month qualitative follow-up and joint interpretation. QUAN, quantitative; qual, qualitative; SDS, Zung Self-Rating Depression Scale.

### 2.2 Participants and recruitment

Registered, on-duty nurses were eligible if they provided direct inpatient psychiatric care, had at least one year of psychiatric nursing experience, could complete the survey and qualitative follow-up, and consented to participate. We excluded nurses working only in outpatient, emergency, administrative or support roles; interns and trainees; nurses reporting a major adverse life event in the preceding three months; those on extended leave or planning to leave; those unable to participate because of cognitive impairment; and those enrolled in another mood-related intervention. Convenience recruitment was used within each hospital. Of 420 electronic questionnaires distributed, 357 passed quality checks and were analyzed (valid response rate, 85.0%).

The original protocol specified a minimum quantitative sample of 352, based on an anticipated prevalence of a positive depressive-symptom screen of 40–50%, two-sided α=0.05, a 0.05 absolute margin of error, a design effect of 1.5 and 15% nonresponse. The 40–50% range was derived from Chinese surveys of hospital nurses that used the Zung SDS and reported depressive-symptom prevalences of approximately 40% (23); because no equivalent estimate was available for psychiatric nurses in northern Jiangsu, the upper end of that range was carried into the calculation, since values closer to 0.5 maximize the required sample size. The achieved sample of 357 exceeded this minimum.

Twenty nurses entered the qualitative follow-up. Purposive selection sought variation in sex, age, experience, ward, hospital and SDS index score. The group comprised 16 women and four men, aged 26–44 years, with 3–20 years of psychiatric nursing experience. Each hospital contributed five participants (**Supplementary Table S5**). Severity labels in the original selection table had been assigned with an alternative cut-off set (≥50 mild, ≥60 moderate, ≥70 severe) rather than the thresholds prespecified for this study; all 20 labels were therefore recomputed from the SDS index score using the prespecified cut-offs (53–62 mild, 63–72 moderate, ≥73 severe), which reclassified six participants. The recomputed subsample comprised seven screen-negative nurses and 13 screen-positive nurses (five mild, five moderate and three severe), and the index score and stratum for each participant are reported in **Supplementary Table S5**. Thematic saturation was reached at participant 17. The three remaining planned interviews were completed to retain representation across sites.

### 2.3 Quantitative measures

#### 2.3.1 Depressive symptoms

The 20-item Zung Self-Rating Depression Scale (SDS) assessed depressive symptoms (24). Raw scores were converted to standard index scores; scores of 53–62, 63–72 and ≥73 were classified as mild, moderate and severe, respectively. A score ≥53 defined a positive screen. The SDS is not a diagnostic interview, and population-specific interpretation remains important in China (25). Cronbach’s alpha for the SDS was 0.87 in the present sample.

#### 2.3.2 Occupational strain

Occupational strain was assessed with the Personal Strain Questionnaire of the Occupational Stress Inventory–Revised (26). The analytical dataset contained standardized T-scores, with higher values indicating greater strain. Cronbach’s alpha for the Personal Strain Questionnaire was 0.91 in the present sample.

#### 2.3.3 Psychological resilience

Psychological resilience was assessed with the 25-item Connor–Davidson Resilience Scale (CD-RISC-25) (27), administered in Chinese. Each item is rated from 0 (“not true at all”) to 4 (“true nearly all the time”) and the 25 item scores are summed, giving a total score of 0–100. Higher scores indicate greater resilience. No linear transformation, rescaling or subscale weighting was applied: the analyses used the raw total in its original units, so the per-point estimates in **Supplementary Table S2** refer to one point of the 0–100 CD-RISC-25 total. A COSMIN review emphasizes that the version and the context should be specified whenever CD-RISC scores are compared (28). The score variable was re-checked against the questionnaire codebook after review and corresponds to the untransformed summed raw score. Cronbach’s alpha for the CD-RISC-25 was 0.89 in the present sample.

#### 2.3.4 Occupational and demographic characteristics

Participants reported age, sex, marital status, education, professional title, psychiatric nursing experience, ward type, hospital location and average monthly night shifts. Workplace violence was assessed using Wang’s Workplace Violence Scale (WVS) (29), with a recall period of the preceding 12 months. The WVS covers three forms of violence: physical, verbal or psychological, and sexual. Because inpatients in these closed wards were not routinely accompanied by relatives, only incidents perpetrated by inpatients, or by family members during visits, were counted. Participants reporting at least one incident of any included form during the preceding 12 months were classified as exposed to workplace violence. Cronbach’s alpha was 0.92 for the original scale and 0.861 in the present sample.

### 2.4 Quantitative data collection and analysis

Hospital nursing departments distributed an anonymous mobile questionnaire that required electronic consent before access and accepted one submission per device and internet protocol address. Attention checks, logic checks and a minimum completion time identified invalid responses, and the analytical dataset contained no missing values. Analyses were performed in R version 4.6.1. Continuous variables are reported as mean (standard deviation [SD]) and categorical variables as n (%). Unadjusted group comparisons used Welch’s t test, Pearson’s chi-square test or Fisher’s exact test, as appropriate. Their P values are exploratory and were not adjusted for multiple comparisons. Pearson coefficients described continuous-variable correlations.

Because 38.7% of participants screened positive, the primary analysis used a modified Poisson generalized linear model with a log link and HC0 robust standard errors to estimate prevalence ratios (PRs). Workplace violence, average monthly night shifts, occupational strain and resilience were entered simultaneously; continuous predictors were standardized so that PRs represent a one-SD increase. Hospital location was included as a fixed effect to absorb between-site differences in casemix, staffing and reporting culture. These four exposures were prespecified from the conceptual model that motivated the study, and for that reason they alone constitute the primary model. Age, sex, education, professional title and ward type were not treated as confounders of primary interest: ward allocation and roster assignment are plausibly determined jointly with, or downstream of, these characteristics, so adjusting for them risks conditioning on part of the exposure pathway, and adding five further terms to a model with 138 events would have reduced precision without answering a prespecified question. They were therefore examined in a prespecified sensitivity model, and a raw-unit model aided interpretation. Exploratory violence-by-resilience and strain-by-resilience terms were evaluated separately; because they were not prespecified, their P values are hypothesis-generating. All tests were two-sided with α=0.05.

### 2.5 Qualitative data collection and analysis

The qualitative follow-up lasted three months. Each nurse completed a monthly one-to-one semistructured interview and a weekly reflective diary; participant observation was conducted in relevant ward contexts. The guide covered emotionally demanding events, professional value, coping, resilience and organizational support. Interviews were non-leading, audio-recorded in full and transcribed verbatim. Transcripts, diaries and observation logs were de-identified and linked by participant code.

Interviews and ward observations were conducted by four members of the research team (Y.H., J.H., Q.W. and Y.T.), all registered nurses with postgraduate qualifications and prior training in qualitative interviewing; Y.H. and Y.T. led the interviews and J.H. and Q.W. conducted the ward observations. Participants were told at consent that the interviewers were acting as researchers rather than as managers, and no nurse was interviewed or observed by a team member holding line-management or performance-appraisal responsibility for them. Each interviewer kept a reflexive journal recording prior assumptions about workplace violence, shift work, professional recognition and organizational support, and these entries were reviewed at fortnightly team meetings; where a researcher’s own ward experience appeared to be shaping a code, the code was re-derived from the transcript by a second analyst who had not conducted that interview. Field notes recorded the setting, non-verbal responses and any interruptions.

Colaizzi’s seven-step descriptive phenomenological method guided analysis: repeated reading, extraction of significant statements, formulation of meanings, theme clustering, exhaustive description, development of a fundamental structure and confirmation or refinement. Coding disagreements were discussed by the research team, and longitudinal comparison distinguished isolated incidents from patterns recurring across follow-up. No new themes arose after participant 17; the three further planned participants were retained to complete cross-site representation.

The three data sources were combined within case before they were compared across cases. For each participant, transcripts, diary entries and observation notes were assembled chronologically into a single case file linked by participant code. Transcripts carried the extraction of significant statements and the formulation of meanings; diaries established whether an experience recurred between interviews or was confined to one event, and so separated repeated strain from isolated incidents; observation notes contextualized accounts of staffing, ward atmosphere and incident handling, and were used to check whether described practices were visible in the setting. Divergence between sources — for example, a diary recording repeated low-intensity threats that a participant had not raised in interview — was retained in the case summary and examined at cross-case level rather than resolved in favor of one source. Meanings were carried into the theme clusters only when they were supported in at least two sources or confirmed with the participant at the following interview.

### 2.6 Mixed-methods integration

Integration linked the phases through sampling, comparison and interpretation. Quantitative screening scores informed purposive qualitative sampling; a joint display compared quantitative estimates with qualitative evidence, convergence or divergence and meta-inferences; and an expert group of two nursing-management experts, two psychology-department directors and two psychiatric head nurses reviewed the interpretive framework. The framework is a synthesis, not a tested causal pathway. Reporting followed STROBE for the quantitative phase, COREQ for the qualitative phase and established mixed-methods reporting principles; the COREQ items on the research team, reflexivity and integration of data sources that were previously incomplete are now reported in Section 2.5.

### 2.7 Ethical considerations

The study was approved by the Ethics Committee of Huai’an Third People’s Hospital (approval no. 2024-01). Participants provided informed consent electronically before the survey and separately for qualitative recording and quotation. Survey records, transcripts and quotations were de-identified, and participants could withdraw without employment consequences.

## 3 Results

### 3.1 Participant characteristics

The sample was predominantly female and mid-career, recruitment was nearly balanced across the four hospitals, and 143 nurses (40.1%) reported workplace violence in the preceding 12 months (**Table 1**). Positive screens were more frequent among nurses exposed to violence (53.6% vs 31.5%) and among those with more night work or greater strain, whose resilience scores were also lower. **Table 1** P values are exploratory and uncorrected, so individual demographic comparisons were not interpreted.

**Table 1:** Participant characteristics by depressive-symptom screening status (N = 357).

Characteristic	Level	Overall	Screen-negative (n=219)	Positive screen (n=138)	P value
Age, years		34.60 (8.26)	34.77 (8.04)	34.34 (8.63)	0.641
Psychiatric nursing experience, years		10.46 (7.64)	10.47 (7.58)	10.43 (7.76)	0.966
Night shifts per month		5.86 (2.93)	5.08 (2.66)	7.09 (2.92)	<0.001
Occupational strain (PSQ T-score)		68.42 (11.37)	65.26 (10.82)	73.44 (10.41)	<0.001
Psychological resilience (CD-RISC total)		62.15 (9.81)	64.89 (9.23)	57.81 (9.14)	<0.001
Hospital location	Huai’an	89 (24.9%)	58 (26.5%)	31 (22.5%)	0.595
	Lianyungang	89 (24.9%)	50 (22.8%)	39 (28.3%)	
	Suqian	89 (24.9%)	57 (26.0%)	32 (23.2%)	
	Xuzhou	90 (25.2%)	54 (24.7%)	36 (26.1%)	
Sex	Female	325 (91.0%)	197 (90.0%)	128 (92.8%)	0.477
	Male	32 (9.0%)	22 (10.0%)	10 (7.2%)	
Marital status	Divorced/widowed	21 (5.9%)	14 (6.4%)	7 (5.1%)	0.035
	Married	251 (70.3%)	163 (74.4%)	88 (63.8%)	
	Unmarried	85 (23.8%)	42 (19.2%)	43 (31.2%)	
Education	Bachelor or above	321 (89.9%)	200 (91.3%)	121 (87.7%)	0.351
	Junior college	36 (10.1%)	19 (8.7%)	17 (12.3%)	
Professional title	Intermediate	146 (40.9%)	92 (42.0%)	54 (39.1%)	0.358
	Junior	132 (37.0%)	84 (38.4%)	48 (34.8%)	
	Senior	79 (22.1%)	43 (19.6%)	36 (26.1%)	
Ward type	Acute	120 (33.6%)	70 (32.0%)	50 (36.2%)	0.252
	Geriatric	50 (14.0%)	26 (11.9%)	24 (17.4%)	
	PICU	42 (11.8%)	29 (13.2%)	13 (9.4%)	
	Rehabilitation	145 (40.6%)	94 (42.9%)	51 (37.0%)	
Workplace violence exposure	Not exposed	214 (59.9%)	150 (68.5%)	64 (46.4%)	<0.001
	Exposed	143 (40.1%)	69 (31.5%)	74 (53.6%)	

Continuous variables are mean (SD); categorical variables are n (%). P values are exploratory, unadjusted and not corrected for multiple comparisons; they are from Welch’s t tests, Pearson’s chi-square tests or Fisher’s exact tests, as appropriate. PSQ, Personal Strain Questionnaire; CD-RISC, Connor–Davidson Resilience Scale; PICU, psychiatric intensive care unit.

### 3.2 Depressive-symptom profile and bivariate correlations

The mean SDS index score was 52.17 (SD 12.08) and 138 nurses (38.7%) screened positive; the distribution across the mild, moderate and severe strata is shown in **Figure 2a**. SDS scores correlated positively with night-shift burden and occupational strain and inversely with resilience, and occupational strain and resilience were themselves inversely correlated (**Figure 2b**; **Supplementary Table S1**).

**Figure 2:**
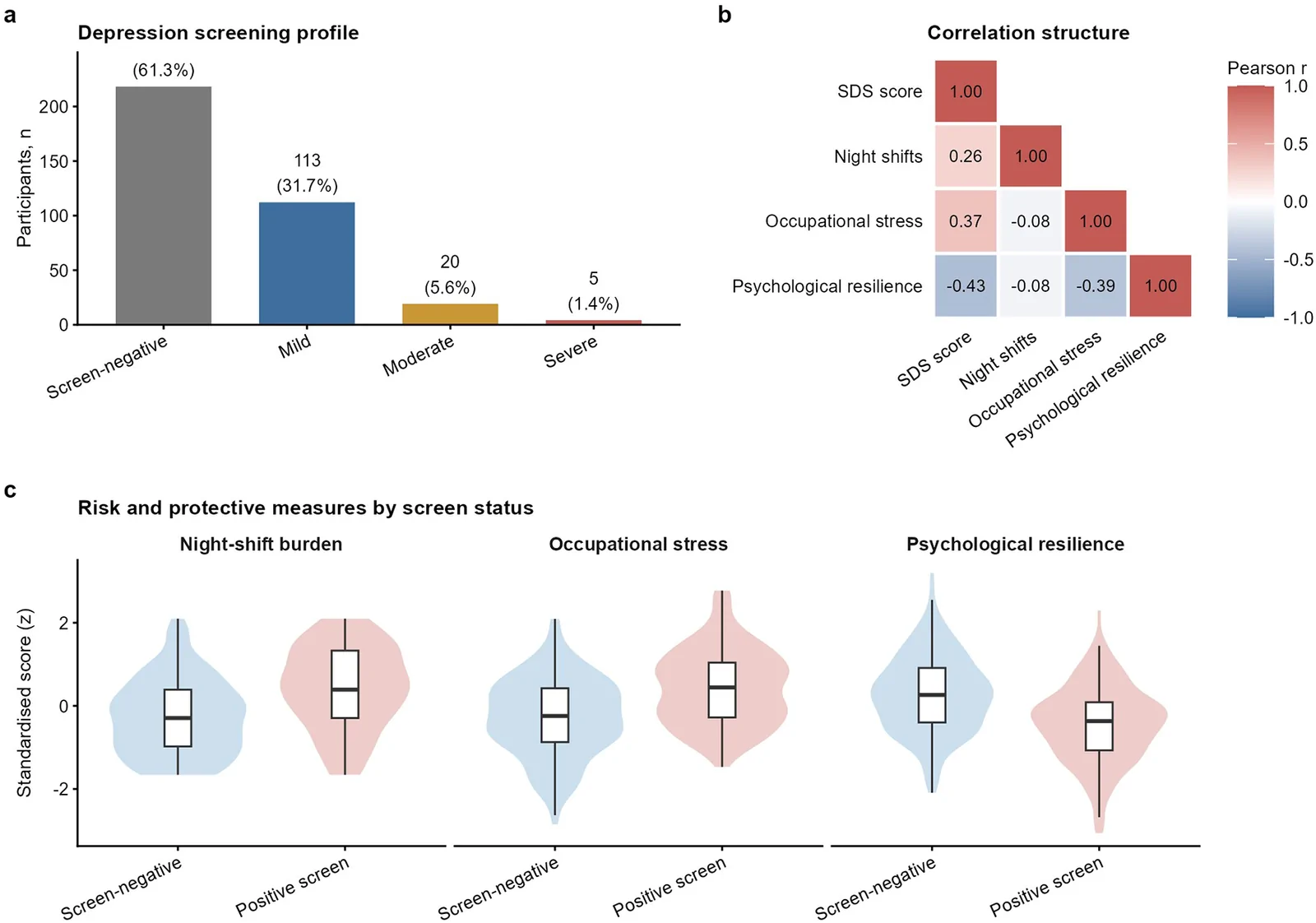
Quantitative profile of depressive symptoms and key exposures. **(a)** SDS screening categories; percentages use the full sample (N = 357) as denominator. **(b)** Pearson correlations among SDS score, night shifts, occupational strain and psychological resilience. **(c)** Distributions of standardized measures by screening status; violin shapes show density, boxes show the interquartile range and center lines show medians. CD-RISC, Connor–Davidson Resilience Scale; PSQ, Personal Strain Questionnaire; SDS, Zung Self-Rating Depression Scale.

### 3.3 Adjusted associations with an SDS-positive screen

Hospital-adjusted estimates showed 65% higher screening prevalence among nurses who reported violence (PR 1.65, 95% CI 1.31–2.08). One SD of additional night-shift burden corresponded to PR 1.48 (95% CI 1.33–1.66). The PR for occupational strain was 1.45 per SD (95% CI 1.28–1.65). Resilience showed a different pattern: PR 0.79 per SD (95% CI 0.70–0.88) (**Table 2**; **Figure 3**). Broader adjustment changed these estimates little (**Supplementary Table S3**). The exploratory violence-by-resilience term was not detectable (P = 0.171). A strain-by-resilience term was detected (P = 0.003), but this *post hoc* result does not support a causal moderation claim (**Supplementary Table S4**).

**Table 2:** Primary modified Poisson model for an SDS-positive screen.

Predictor	Adjusted PR	95% CI	P value
Workplace violence exposure (yes vs no)	1.65	1.31–2.08	<0.001
Night-shift burden (per 1-SD increase)	1.48	1.33–1.66	<0.001
Occupational strain (per 1-SD increase)	1.45	1.28–1.65	<0.001
Psychological resilience (per 1-SD increase)	0.79	0.70–0.88	<0.001

The model included all four predictors simultaneously and hospital as a fixed effect. Continuous predictors were standardized. HC0 robust standard errors were used. PR, prevalence ratio; CI, confidence interval; SDS, Zung Self-Rating Depression Scale.

**Figure 3:**
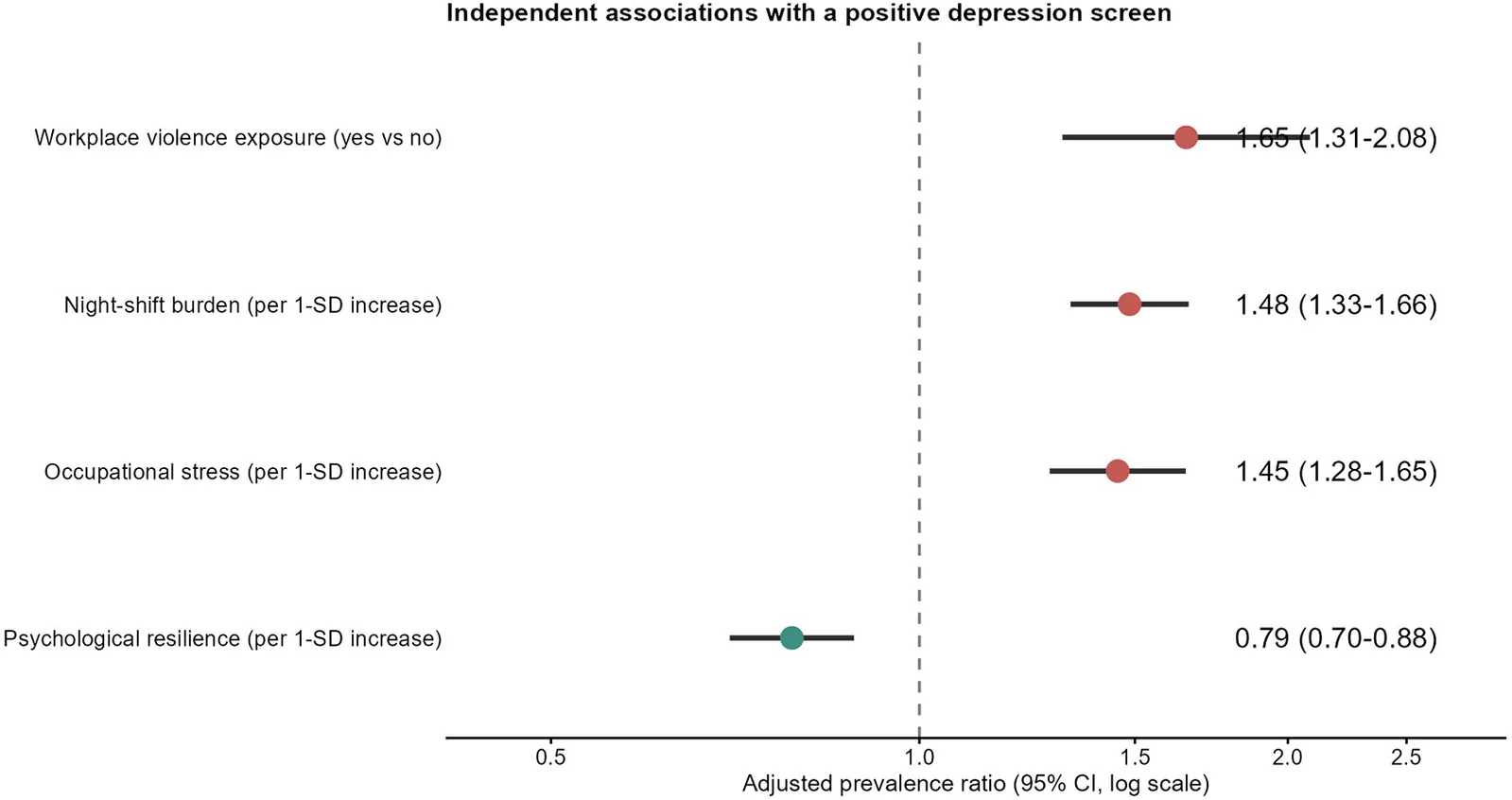
Adjusted associations with an SDS-positive screen. Points are prevalence ratios and horizontal lines are 95% robust confidence intervals from the modified Poisson model with hospital fixed effects. Continuous predictors are expressed per one-standard-deviation increase. The dashed vertical line denotes PR = 1. SDS, Zung Self-Rating Depression Scale.

### 3.4 Qualitative findings

Four themes described how occupational exposures acquired meaning across the three-month follow-up. The phrase repeated occupational strain refers here to recurring low-intensity threats, vigilance and emotional residue; it is an interpretive label rather than a diagnostic construct. Themes did not map one-to-one onto model predictors (**Table 3**).

**Table 3:** Joint display integrating quantitative estimates and qualitative evidence.

Theme	Quantitative result	Qualitative evidence	Relation	Meta-inference
Repeated occupational strain	Violence: PR 1.65 (1.31–2.08); night shifts: PR 1.48 (1.33–1.66)	Threats, verbal aggression and vigilance recurred; N4 described anticipatory anxiety without one major incident.	Convergence	Exposure categories may understate recurring low-intensity events; reporting and follow-up should include them.
Value–recognition mismatch	Occupational strain: PR 1.45 (1.28–1.65)	N17 described social devaluation as harder than workload despite commitment to patient care.	Complementarity	Recognition and anti-stigma measures may address meanings not captured by a strain score.
Unequal recovery resources	Resilience: PR 0.79 (0.70–0.88)	N2 absorbed patient irritability; N8 and N18 described meaning, boundaries and recovery.	Convergence with nuance	Resilience is protective in association, but should not substitute for safer working conditions.
Generic, unresponsive support	No direct quantitative measure	N1 and N17 contrasted group activities with a need for timely, individualized post-incident help.	Expansion	Add responsive support as an organizational target for future measurement and intervention.

PRs are adjusted for the other prespecified predictors and hospital fixed effects. Integration is interpretive and does not establish mediation, direction or causation. PR, prevalence ratio.

#### 3.4.1 Theme 1: repeated occupational strain

Repeated low-intensity threats, verbal aggression and anticipatory vigilance were distressing even without a single catastrophic event. One nurse said, “There was no major incident, but before every shift I would automatically feel anxious and expect something bad to happen. It gradually affected my life, and even things I used to enjoy stopped feeling enjoyable” (N4). Another reported dreaming about patients’ cries after repeated exposure to crying, abuse and non-cooperation (N14). These accounts located burden in repetition and expectation as well as discrete incidents.

#### 3.4.2 Theme 2: professional value–recognition mismatch

Nurses derived meaning from patient care but felt that the complexity and therapeutic value of psychiatric nursing were poorly recognized. One participant stated: “We do everything we can to care for patients, yet people still call us ‘nurses who look after mad people’. Sometimes not being understood is harder than the workload itself” (N17). The mismatch between internal professional commitment and external recognition amplified emotional strain and associative stigma.

#### 3.4.3 Theme 3: layered differences in resilience

Capacity to set emotional boundaries, reinterpret difficult encounters and recover between shifts varied. N2 said, “I feel I cannot solve anything. When patients become irritable, I become irritable too. Every day feels painful and helpless.” By contrast, N8 treated “every small improvement” as evidence that the work had value, while N18 said she rarely carried work emotions home and could gain fulfilment from nursing. These contrasts supported resilience as a protective correlate while showing that recovery resources were uneven.

#### 3.4.4 Theme 4: support perceived as generic and unresponsive

Several nurses knew that support programs existed, yet they did not regard them as useful after a difficult event. N1 recalled group lectures but little individual contact after violence. N17 viewed the annual Nurses’ Day salon as tokenistic because staff needs were not heard. What participants asked for was more immediate: confidential contact, trained counselling, protected debriefing time and follow-up outside performance management.

### 3.5 Integrated findings

Survey and qualitative findings overlapped, but not completely. Violence, night work and strain tracked with screening status; nurses described vigilance, poor recovery and devaluation in concrete terms. Resilience appeared in their accounts as boundary-setting and meaning-making. Organizational support emerged only from the qualitative strand because the survey had not measured it. **Figure 4** therefore links the domains without assigning temporal or causal direction.

**Figure 4:**
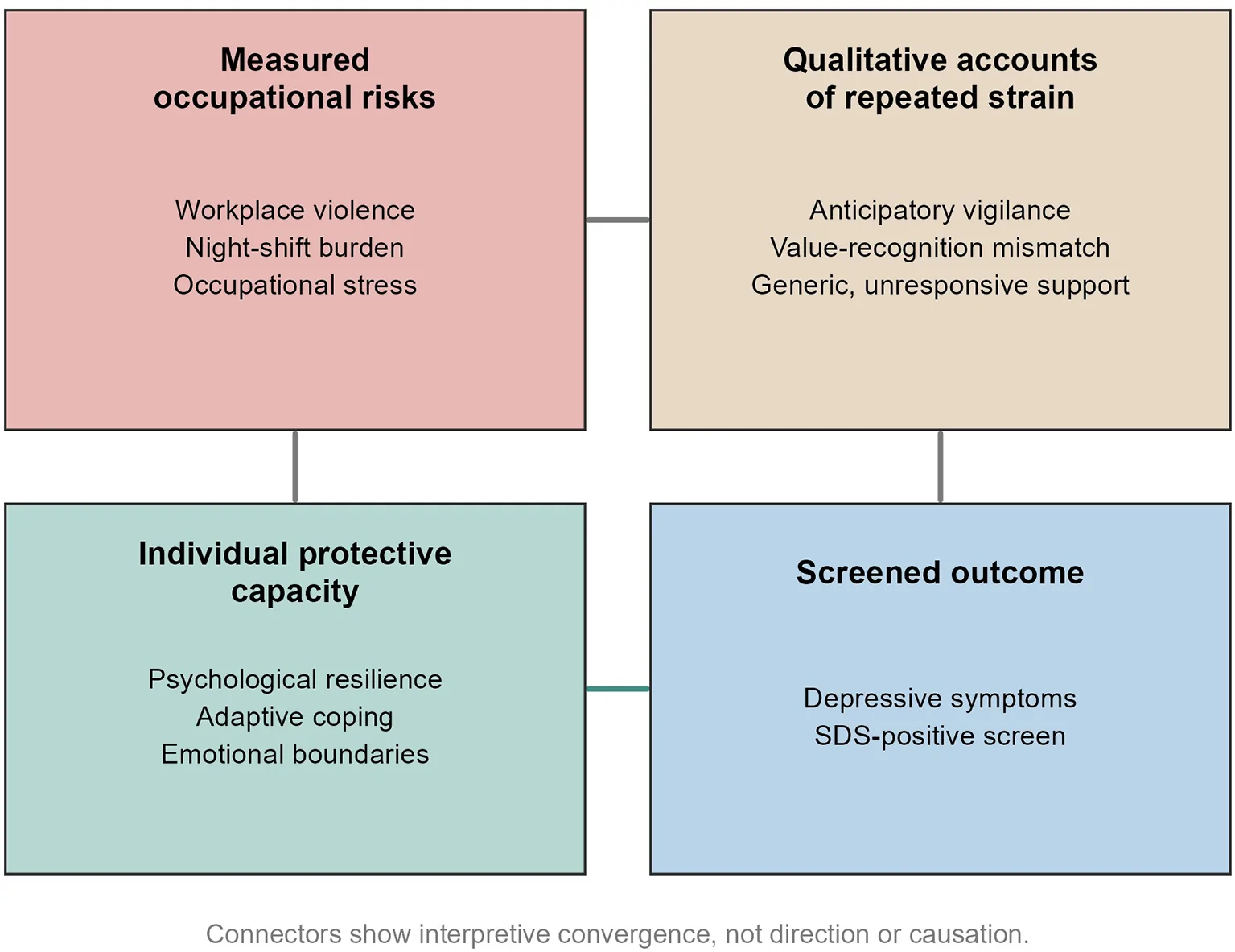
Integrated mixed-methods interpretive framework. Measured occupational risks, qualitative accounts of repeated strain, individual protective capacity and the screened outcome are connected without directional arrows. Teal identifies the protective-domain connection; gray lines show other interpretive connections. The figure does not represent a tested causal pathway. SDS, Zung Self-Rating Depression Scale.

## 4 Discussion

### 4.1 Principal findings

An SDS-positive screen was recorded for 38.7% of the sample. Violence, more night work and greater occupational strain each corresponded to higher prevalence after hospital adjustment. Higher resilience scores corresponded to lower prevalence. Interviews added a less tidy account of the same problem: ordinary threats, lost recovery time, devaluation and poorly timed support often overlapped. This combination argues against relying on coping programs as the sole response.

### 4.2 Interpretation in relation to previous evidence

An SDS-positive screen is not a diagnosis. The 38.7% estimate therefore describes screening burden. Reviews of mental health nursing have documented burnout, compassion fatigue and secondary traumatic stress (1, 2), and a survey of 64 hospitals in six European countries found high burnout in about a quarter of nurses, with better work environments and staffing associated with fewer adverse clinician outcomes (30). A meta-analysis across nursing samples also found a substantial association between burnout and depression (5). Their samples and measurements differ. Prevalence should not be compared directly across instruments, thresholds, settings or study periods.

Recent evidence links workplace violence to adverse psychological outcomes. A scoping review identified anxiety, fear, post-traumatic stress and depression after exposure (6). A multicenter longitudinal study of Chinese nurses also associated workplace violence with subsequent depression and anxiety (8), and Italian mental health nurses interviewed after assault described altered work habits and heightened caution rather than discrete symptom episodes (7). Our interviews show why a binary exposure indicator is incomplete. Verbal hostility, near misses and anticipation of danger mattered even when no serious injury occurred. Post-incident systems limited to major assaults would miss those experiences.

Night work was related to screening status in the survey. A meta-analysis of nurses reported higher odds of depression among night-shift workers (OR 1.49, 95% CI 1.26–1.76) (11), close in magnitude to the per-SD prevalence ratio observed here, and sleep problems are associated with burnout in nursing populations (12). On psychiatric wards, circadian disruption may coincide with lean staffing and prolonged vigilance. Fewer colleagues may also be available for immediate support. Our data cannot separate these pathways, but roster intensity and recovery time can be changed.

Higher resilience scores accompanied lower screening prevalence, consistent with studies linking resilience to better psychological outcomes in mental health nurses (16, 17). Intervention research suggests that resilience training or mindfulness can improve resilience and some work-related outcomes (18, 19). Participants gave the construct practical meaning: keeping emotional boundaries, recovering after shifts and noticing small gains in patient care. Narrative work with mental health nurses locates resilience in the same places — proactive management of the professional self, supportive relationships and active self-care — rather than in individual stoicism (31). The *post hoc* strain-by-resilience term warrants study, but it does not demonstrate causal moderation. Working conditions and access to support remain organizational responsibilities, and reviews support workplace-level changes rather than sole reliance on individual programs (20–22).

### 4.3 What the qualitative phase explained beyond the survey

The survey estimated how strongly four exposures tracked with screening status. It could not describe what those exposures consisted of, why nurses with similar scores differed, or what the model had left out. The qualitative phase addressed each of these, and three of its contributions are specific enough to be stated as findings rather than as illustration.

First, it re-specified the exposure. Workplace violence entered the model as a 12-month binary indicator anchored on discrete incidents, yet what nurses described as burdensome was the accumulation of low-intensity threat and the vigilance between incidents (N4, N14). A nurse with no codeable incident may still carry continuous anticipatory load. This is a measurement problem the survey cannot detect: it would bias a binary indicator toward the null, and it explains why post-incident systems triggered by serious assault cannot reach the exposure that nurses actually reported.

Second, it identified what the resilience score was standing for. CD-RISC-25 yields a single number, but the accounts distinguished at least three capacities behind it: setting emotional boundaries at the end of a shift (N18), reinterpreting difficult encounters as evidence of clinical value (N8) and, where neither was available, absorbing patients’ affect without recovery (N2). The inverse association is therefore compatible with several mechanisms, and reading a low score as a training deficit is only one of them.

Third, it identified a domain the survey had omitted. Organizational support was not measured in the quantitative phase, yet it was what nurses raised most consistently when asked what would have changed the outcome of a difficult shift, and their objection was to the timing and specificity of support rather than to its existence (N1, N17). This is an expansion rather than a convergence result: it generates the hypothesis that the responsiveness of support, not its availability, is the modifiable variable, and it names the construct that the next survey in this population should measure.

The integration was asymmetric in the other direction as well. Night-shift burden carried a per-SD prevalence ratio comparable to that for occupational strain, but nurses discussed rosters mainly as a background condition, and the interview guide did not probe recovery time, commuting or domestic load separately. We therefore treat night work as quantitatively supported but qualitatively under-examined, and we do not claim that the qualitative phase explains that association.

### 4.4 Implications for policy and practice

Two kinds of statement are distinguished here. Exposure to violence, night-shift burden and occupational strain were each associated with screening status in this sample, so measures that reduce them are indicated by our own findings; how best to reduce them is not established here, and the specific measures named below are drawn from external evidence and from participants’ own suggestions rather than from the variables we assessed. Staffing, environmental safety and de-escalation resources may reduce exposure, although trial evidence from acute and forensic settings remains limited (9), and international reviews of prevention programs converge on multicomponent approaches combining training, post-incident debriefing, rapid response teams and environmental modification (10, 32). Rosters should avoid concentrating night shifts without adequate recovery (11, 21). Confidential routine screening and prompt post-incident contact, debriefing and trauma-informed assessment were what participants asked for and are consistent with that external evidence, but no screening, debriefing or post-incident program was measured in this study, so statements about their effect on depressive symptoms are extrapolations rather than results.

Voluntary resilience or mindfulness programs may add value (18, 19). They should, however, accompany rather than substitute for changes in working conditions (20, 21); in the six-country European survey cited above, clinicians themselves ranked better staffing and less bureaucracy ahead of individual mental-health interventions (30). Participants were clear that annual lectures and generic activities were not enough. Regular clinical supervision and confidential counselling may offer more direct support (2, 33), although neither was assessed in this study. Recognition of psychiatric nursing expertise may also reduce the value–recognition gap (14, 15).

### 4.5 Strengths and limitations

Four hospitals contributed complete survey data. Modified Poisson regression estimated prevalence ratios and accounted for site. The qualitative follow-up lasted three months and combined interviews, diaries and observation, avoiding dependence on a single retrospective interview. Reading the estimates beside the narratives also revealed where the two strands did not overlap.

Several issues limit interpretation. Convenience recruitment from four tertiary hospitals restricts transferability. The cross-sectional survey cannot establish temporal order, and all main measures were self-reported. Item-level responses were not retained in the analytical dataset, so the internal-consistency coefficients reported in Section 2.3 could not be re-derived at item level; the CD-RISC-25 version, its 0–100 scoring and the absence of any transformation have, however, been verified against the score distribution and the questionnaire codebook. The 20-person follow-up may not capture longer trajectories. Unmeasured sleep, staffing and prior mental health may confound the estimates. The qualitative themes and integrated framework have not been externally validated.

Two directions follow from these limitations. The occupational dimensions examined here may not arise independently of the clinical work itself: greater occupational strain, more demanding shifts and fewer opportunities for recovery may partly reflect the complexity of the patients being cared for and the intensity of the nursing care they require, which we did not measure. Future studies could complement self-reported occupational measures with routinely collected nursing data, including information extracted and standardized from unstructured electronic nursing documentation through natural language processing, to better characterize the clinical work context associated with nurses’ psychological outcomes (34).

Future studies should move beyond conventional workload indicators and examine whether nursing complexity represents a hidden upstream determinant of occupational strain and depressive symptoms. The psychological burden attributed to demanding shifts may, in part, originate from the intensity, heterogeneity, and accumulation of patients’ nursing care needs. Integrating standardized nursing languages and Nursing Minimum Data Sets would make this otherwise largely invisible dimension measurable through nursing diagnoses and nursing activities, allowing researchers to determine whether the complexity of care itself contributes independently to nurses’ occupational and psychological burden (35–42).

## 5 Conclusion

An SDS-positive screen was common in this sample of psychiatric nurses. Violence, night work and occupational strain were linked to higher prevalence, while resilience showed an inverse relation. Nurses’ accounts located these associations in repeated vigilance, devaluation and support that did not match the moment of need. Safer work systems and responsive post-incident care should precede, not be replaced by, individual resilience programs. The study does not establish causal effects.

## Data Availability

The original contributions presented in the study are included in the article/**Supplementary Material**. Further inquiries can be directed to the corresponding author.

## Supplementary material

Supplementary Table 1 Pearson correlations among continuous study measures (N = 357). SDS, Zung Self-Rating Depression Scale.

Supplementary Table 2 Modified Poisson model expressed in original measurement units. All four predictors and hospital fixed effects were included simultaneously; HC0 robust standard errors were used. CD-RISC values are expressed per one point of the 0–100 CD-RISC-25 total, which was analyzed without transformation. PR, prevalence ratio; PSQ, Personal Strain Questionnaire; CD-RISC, Connor–Davidson Resilience Scale.

Supplementary Table 3 Sensitivity model for an SDS-positive screen. The sensitivity model additionally adjusted for age, sex, education, professional title and ward type; hospital remained a fixed effect. HC0 robust standard errors were used. Only the four prespecified primary predictors are displayed. PR, prevalence ratio.

Supplementary Table 4 Exploratory interaction tests. Interaction terms were *post hoc*. The strain-by-resilience term was statistically detectable, but no causal moderation claim is made.

Supplementary Table 5 Characteristics of qualitative follow-up participants (N = 20). The SDS index score is the raw score multiplied by 1.25 and rounded to an integer. Screening strata follow the thresholds prespecified for this study: <53 no depressive symptoms, 53–62 mild, 63–72 moderate, ≥73 severe. Labels in the original selection table had been assigned with an alternative cut-off set (≥50, ≥60, ≥70) and have been recomputed here; six participants (N6, N8, N11, N13, N16 and N19) changed stratum. SDS, Zung Self-Rating Depression Scale.
