# Supplementary material for: Depressive symptoms and occupational correlates among psychiatric nurses in northern Jiangsu: an explanatory sequential mixed-methods study
Source: Front Psychiatry. 2026 Oct 21;17:1967611. doi: 10.3389/fpsyt.2026.1967611 (PMC13730849; doi:10.3389/fpsyt.2026.1967611)
Supplement: Supplementary file 1 [file SupplementaryFile1.docx]

# **Supplementary material**

The supplementary analyses below are provided for transparent interpretation of the main model. They do not change the primary conclusions.

**Supplementary Table S1.** Pearson correlations among continuous study measures (N=357)

| **Measure** | **SDS score** | **Night shifts** | **Occupational strain** | **Psychological resilience** |
| --- | --- | --- | --- | --- |
| SDS score | 1.000 | 0.258 | 0.366 | -0.430 |
| Night shifts | 0.258 | 1.000 | -0.077 | -0.075 |
| Occupational strain | 0.366 | -0.077 | 1.000 | -0.390 |
| Psychological resilience | -0.430 | -0.075 | -0.390 | 1.000 |

*Note.* SDS, Zung Self-Rating Depression Scale.

**Supplementary Table S2.** Modified Poisson model expressed in original measurement units

| **Predictor** | **Adjusted PR** | **95% CI** | **P value** |
| --- | --- | --- | --- |
| Workplace violence exposure (yes vs no) | 1.652 | 1.309–2.085 | <0.001 |
| Night shifts (per additional shift/month) | 1.145 | 1.102–1.189 | <0.001 |
| Occupational strain (per PSQ T-score point) | 1.033 | 1.022–1.045 | <0.001 |
| Psychological resilience (per CD-RISC point) | 0.976 | 0.964–0.988 | <0.001 |

*Note.* All four predictors and hospital fixed effects were included simultaneously; HC0 robust standard errors were used. CD-RISC values are expressed per one point of the 0–100 CD-RISC-25 total, which was analysed without transformation. PR, prevalence ratio; PSQ, Personal Strain Questionnaire; CD-RISC, Connor–Davidson Resilience Scale.

**Supplementary Table S3.** Sensitivity model for an SDS-positive screen

| **Primary predictor** | **Adjusted PR** | **95% CI** | **P value** |
| --- | --- | --- | --- |
| Workplace violence exposure (yes vs no) | 1.66 | 1.31–2.10 | <0.001 |
| Night-shift burden (per 1-SD increase) | 1.50 | 1.34–1.68 | <0.001 |
| Occupational strain (per 1-SD increase) | 1.46 | 1.29–1.65 | <0.001 |
| Psychological resilience (per 1-SD increase) | 0.78 | 0.70–0.88 | <0.001 |

*Note.* The sensitivity model additionally adjusted for age, sex, education, professional title and ward type; hospital remained a fixed effect. HC0 robust standard errors were used. Only the four prespecified primary predictors are displayed. PR, prevalence ratio.

**Supplementary Table S4.** Exploratory interaction tests

| **Interaction** | **P value** |
| --- | --- |
| Violence exposure x resilience | 0.171 |
| Occupational strain x resilience | 0.003 |

*Note.* Interaction terms were post hoc. The strain-by-resilience term was statistically detectable, but no causal moderation claim is made.

**Supplementary Table S5.** Characteristics of qualitative follow-up participants (N=20)

| **ID** | **Sex** | **Age, years** | **Experience, years** | **Ward** | **Hospital** | **SDS index score** | **Screening stratum** |
| --- | --- | --- | --- | --- | --- | --- | --- |
| N1 | Female | 28 | 5 | Rehabilitation | Xuzhou | 42 | None |
| N2 | Female | 35 | 10 | Acute | Lianyungang | 45 | None |
| N3 | Male | 41 | 18 | PICU | Huai'an | 38 | None |
| N4 | Female | 32 | 8 | Older adult | Suqian | 47 | None |
| N5 | Female | 26 | 3 | Rehabilitation | Xuzhou | 44 | None |
| N6 | Female | 30 | 7 | Acute | Lianyungang | 52 | None |
| N7 | Female | 38 | 15 | Older adult | Huai'an | 55 | Mild |
| N8 | Male | 33 | 9 | PICU | Suqian | 50 | None |
| N9 | Female | 29 | 5 | Acute | Xuzhou | 57 | Mild |
| N10 | Female | 36 | 12 | Rehabilitation | Lianyungang | 53 | Mild |
| N11 | Female | 34 | 10 | PICU | Huai'an | 62 | Mild |
| N12 | Female | 40 | 17 | Acute | Suqian | 65 | Moderate |
| N13 | Female | 31 | 8 | Rehabilitation | Xuzhou | 61 | Mild |
| N14 | Male | 37 | 13 | Older adult | Lianyungang | 67 | Moderate |
| N15 | Female | 29 | 6 | PICU | Huai'an | 63 | Moderate |
| N16 | Female | 33 | 11 | Acute | Suqian | 71 | Moderate |
| N17 | Female | 39 | 16 | PICU | Xuzhou | 75 | Severe |
| N18 | Female | 44 | 20 | Older adult | Lianyungang | 73 | Severe |
| N19 | Male | 36 | 12 | Acute | Huai'an | 70 | Moderate |
| N20 | Female | 42 | 19 | PICU | Suqian | 78 | Severe |

*Note. The SDS index score is the raw score multiplied by 1.25 and rounded to an integer. Screening strata follow the thresholds prespecified for this study: <53 no depressive symptoms, 53–62 mild, 63–72 moderate, ≥73 severe. Labels in the original selection table had been assigned with an alternative cut-off set (≥50, ≥60, ≥70) and have been recomputed here; six participants (N6, N8, N11, N13, N16 and N19) changed stratum. SDS, Zung Self-Rating Depression Scale.*
